# Longitudinal ctDNA Monitoring for Postsurgical Disease Surveillance in Patients with Stage I to IIIB Melanoma

**DOI:** 10.1158/1078-0432.CCR-25-3643

**Published:** 2026-02-03

**Authors:** George Ansstas, Karam Khaddour, Sumedha Sudhaman, Karen Lin, Griffin L. Budde, Andrew Poklepovic, Bently P. Doonan, Meghan J. Mooradian, Ryan J. Sullivan, Vincent T. Ma, Jeremy Rosiecki, Steven Liu, Ronald Drengler, Daniel B. Flora, Georgia M. Beasley, Kristen E. Rhodin, John R. Hyngstrom, Elise K. Brunsgaard, J. Bryce Ortiz, Giby V. George, Michael Krainock, Minetta C. Liu, Alan Tan

**Affiliations:** 1 Washington University in Saint Louis, St. Louis, Missouri.; 2 Dana-Farber Cancer Institute, Harvard Medical School, Boston, Massachusetts.; 3 Natera, Inc., Austin, Texas.; 4 VCU Massey Comprehensive Cancer Center, Richmond, Virginia.; 5 University of Florida, Gainesville, Florida.; 6 Massachusetts General Hospital, Boston, Massachusetts.; 7 University of Wisconsin-Madison, Madison, Wisconsin.; 8 Alaska Oncology and Hematology, Alaska Regional Hospital, Anchorage, Alaska.; 9 The START Center for Cancer Care, San Antonio, Texas.; 10 St Elizabeth Cancer Center, Edgewood, Kentucky.; 11 Duke University Medical Center, Durham, North Carolina.; 12 Rush University Medical Center, Chicago, Illinois.

## Abstract

**Purpose:**

Circulating tumor DNA (ctDNA) has emerged as an important biomarker for early recurrence detection and disease status monitoring during treatment in patients with cancer, including melanoma. We evaluate the prognostic value and impact of postoperative ctDNA detection in patients with stage I to IIIB melanoma using a clinically validated ctDNA assay.

**Experimental Design:**

We conducted a retrospective analysis of real-world data of patients with stage I to IIIB melanoma, including ctDNA results using a personalized, tumor-informed, 16-plex multiplex PCR–next-generation sequencing assay. Adjuvant treatment decisions and postsurgical plasma sample collection timepoints were at the physician’s discretion. ctDNA results were correlated with clinical outcomes.

**Results:**

Across 190 patients and a total of 1,578 samples, a median of 7 ctDNA tests (range: 1–33) per patient were performed over a median period of 24.6 months (range: 3.7–74.7). ctDNA positivity at any postoperative timepoint was significantly associated with shorter recurrence-free survival [RFS; hazard ratio (HR): 40.63; 95% confidence interval (CI), 19.9–82.96; *P* < 0.0001). This finding was also observed in patients specifically with regional or distant recurrence (HR: 39.55; 95% CI, 18.08–86.51; *P* < 0.0001). In multivariate analysis, ctDNA positivity was the most significant prognostic factor associated with RFS when compared with other clinicopathologic factors, including stage, sex, and mitotic rate (HR: 25.36; 95% CI, 9.16–70.3; *P* < 0.001).

**Conclusions:**

Our findings highlight the prognostic value of postsurgical, personalized ctDNA detection of recurrence and longitudinal disease surveillance in stage I to IIIB melanoma. The impact of ctDNA on real-world clinical decision-making highlights the need to assess outcomes when cancer management is influenced by ctDNA dynamics.

**Translational Relevance**

Patients with resected stage I to IIIB melanoma remain at risk for recurrence despite advances in adjuvant therapy, and current clinicopathologic staging alone does not reliably identify who will benefit most from treatment. Circulating tumor DNA (ctDNA) has emerged as a promising biomarker to detect disease recurrence and guide management across cancers. In this large, real-world analysis, postsurgical ctDNA positivity was strongly and independently associated with poor recurrence-free survival, emerging as the most significant prognostic factor compared with traditional clinicopathologic features. Notably, ctDNA positivity was also associated with subsequent changes in clinical management, including intensified imaging or initiation or modification of systemic therapy, in some cases preceding radiographic evidence of disease. These findings demonstrate that longitudinal ctDNA monitoring provides powerful prognostic information and is associated with meaningful changes in care for early-stage melanoma. Prospective studies are now warranted to determine whether ctDNA-guided management improves patient outcomes.

## Introduction

In recent years, significant advances have improved survival outcomes in patients with melanoma. Notably, immune checkpoint inhibitors (ICI), including anti–programmed cell death 1 (PD-1) and/or anti–cytotoxic T cell–associated antigen 4 (CTLA-4) therapy, as well as BRAF–MEK inhibitors, have substantially improved survival rates among patients with advanced melanoma (1–5). In addition, clinical trials of adjuvant ICI in stage II to III melanoma have shown improved recurrence-free survival (RFS). In the CheckMate 76K and KEYNOTE-716 trials, patients with stage IIB and IIC melanoma experienced RFS benefits with ICI therapy compared with placebo (6, 7). Despite these advances, there are insufficient data on the overall survival (OS) benefit with adjuvant ICI guided by staging information alone, which highlights the need to investigate other prognostic factors that could guide adjuvant treatment decisions and improve clinical outcomes.

Clinical assessment and radiographic imaging, including cross-sectional CT and whole-body 2[^18^F]fluoro-2-deoxy-D-glucose PET/CT and/or MRI, remain the mainstay of surveillance per National Comprehensive Cancer Network (NCCN) guidelines (8). However, for malignant melanoma, routine surveillance imaging is reserved only for those with more advanced disease. According to the American Joint Committee on Cancer (AJCC) staging system (eighth edition), tumor thickness and ulceration continue to be important prognostic factors alongside regional lymph node involvement among patients with melanoma (9, 10). Previously, tumor proliferation as inferred from the mitotic rate was also included among the high-risk factors for recurrence, but it was removed as a T1-category criterion in the eighth edition of the AJCC melanoma staging system (College of American Pathologists cancer template; ref. 10). Despite the global acceptance of the AJCC staging system, it has notable limitations. For example, patients with stage IIB/IIC melanoma often exhibit worse survival compared with those with stage IIIA disease, which paradoxically suggests a more advanced stage. This discrepancy highlights the need for new prognostic biomarkers that could help with accurate risk stratification (10). To this end, ctDNA has emerged as a promising tool for assessing molecular residual disease (MRD) across various malignancies (11–15) and has demonstrated encouraging results in preliminary melanoma studies (1, 16–21).

Here, we evaluated the prognostic value of postsurgical ctDNA detection in patients with stage I to IIIB melanoma using a personalized, tumor-informed ctDNA assay (Signatera, Natera, Inc.). We report ctDNA positivity rates measured longitudinally and their association with RFS. We provide data comparing the correlation of RFS with ctDNA status versus other commonly used clinicopathologic prognostic factors. In addition, we illustrate the impact of ctDNA on clinical management in patients with melanoma in a real-world setting.

## Materials and Methods

### Subjects and study design

This study was a retrospective analysis of real-world data from prospectively collected longitudinal plasma samples of 197 patients with stage I to IIIB cutaneous melanoma, who were treated at 11 participating institutions between March 2018 and December 2024. Clinicopathologic information was collected for all patients. Of the 197 patients, 7 did not have ctDNA testing following surgery and were excluded from postsurgical and post–definitive treatment surveillance RFS analyses. The timing of postoperative ctDNA collections was at the discretion of the treating physician and was not standardized across sites, and the first postoperative blood draw varied widely (range: 0–60.83 months).

Because this analysis was based on real-world, clinically ordered ctDNA testing, treating physicians had access to ctDNA results in real time and could use them to inform management decisions at their discretion. There was no prespecified study protocol dictating interventions based on ctDNA status. For patients whose management was altered following ctDNA positivity, this determination was based on explicit chart documentation or clinician notes linking ctDNA results to subsequent imaging, treatment initiation, or therapy modification. In cases in which documentation was indirect, the timing of clinical events relative to ctDNA results was reviewed to confirm temporal association.

**Figure 1.**
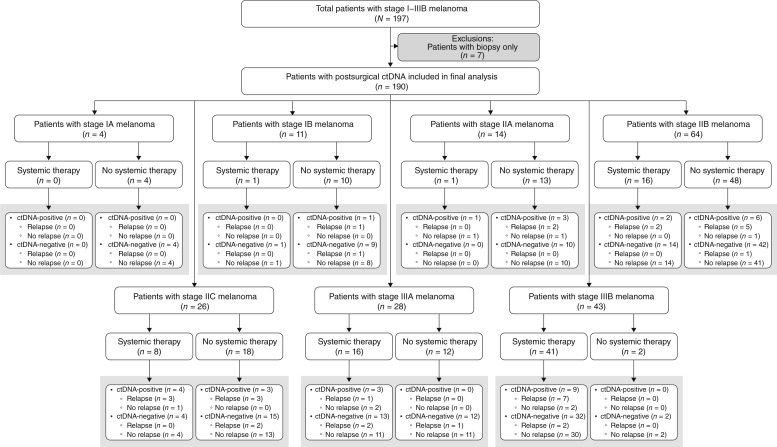
Flow diagram depicting the number of patients included in our study. Patient number is further stratified by stage, receipt of systemic adjuvant treatment or not, and ctDNA outcomes, followed by the number of patients who relapsed or not.

For longitudinal analysis, patients (stage I–IIIB melanoma) were included if they had at least one postsurgical ctDNA test result, available clinically annotated data, confirmed clinical stage, and documented date of surgery (Fig. 1). All timepoints prior to or within 2 weeks following recurrence (based on clinical examination, imaging, and/or pathological confirmation) were included in the analysis. The post–definitive treatment surveillance analysis included timepoints after surgery (if no adjuvant treatment was given) or after completion of adjuvant treatment, until the last follow-up or clinical recurrence. All clinical data were gathered and interpreted by the respective clinical teams at each of the academic institutions, and the de-identified data were shared with Natera, Inc. for analysis. The study was approved by the corresponding Ethical and Independent Review Services and was conducted in accordance with the Declaration of Helsinki. Retrospective analysis of de-identified data, ctDNA results, and clinicopathologic data collected for quality assurance purposes under 45 CFR 164.501 was determined to be exempt research by an independent institutional review board (Salus #20099 - 04). A waiver of the consent process and of the requirement for documentation of informed consent was granted according to 45 CFR 46.116(d) and 45 CFR 46.117(c)(2), respectively.

### Personalized ctDNA assay workflow

For all patients, blood was collected in two 10-mL Streck tubes. All biological specimens were processed following a Clinical Laboratory Improvement Amendments–validated standard operating procedure at Natera, Inc. ctDNA analysis was performed using a clinically validated, personalized, and tumor-informed 16-plex PCR assay (Signatera) in patients through commercial ordering, as previously described (11).

In short, whole-exome sequencing (WES) was performed on formalin-fixed, paraffin-embedded tumor tissue and matched normal DNA blood samples obtained from each patient. Based on the WES results, multiplex PCR (mPCR) primers were designed to identify and track up to 16 patient-specific and tumor-specific somatic single-nucleotide variants (SNV) in the associated patient’s plasma. The mPCR primers targeting the personalized SNVs were used to track ctDNA in the respective patients’ plasma cell-free DNA. Samples with ≥2 SNVs were considered ctDNA-positive, and the ctDNA concentration (levels) was reported as mean tumor molecules (MTM) per mL of plasma. Collection timepoints of ctDNA varied and were per the investigator's discretion.

### Statistical analysis

The primary endpoint was RFS; the duration of RFS was defined as the time from surgery to the date of recurrence or death from any cause/end of RFS event-free follow-up. Patient characteristics were summarized using descriptive statistics, and statistical significance was evaluated using Fisher’s exact test for categorical variables. Survival analyses were conducted using R software v4.2.2 (RRID: SCR_001905) using packages *survminer* (v0.4.9) and *survival* (v3.2.13). Survival curves were compared using the Kaplan–Meier method. Hazard ratios (HR), associated 95% confidence intervals (CI), and *P* values were calculated using Cox regression analysis (R packages *survminer* v0.4.9 and *survival* v3.2.13). A Cox proportional hazards model with a time-dependent covariate was applied. To account for variable timing of postoperative blood draws, ctDNA status was modeled as a time-varying covariate in Cox regression analyses to help ensure that patients contributed risk time under their observed ctDNA status at each timepoint. Lead time was defined as the interval between ctDNA detection and imaging-confirmed recurrence. To account for transient ctDNA fluctuations, we performed a sensitivity analysis defining lead time as the period from the last ctDNA-positive result prior to recurrence, excluding intervening negative samples. A log-rank test was used for comparing two survival distributions, with *P* ≤ 0.05 being considered significant. Multivariate regression models were generated for RFS, performed only for patients who had data points available for all the variables included.

## Results

### Patient cohort

**Figure 2.**
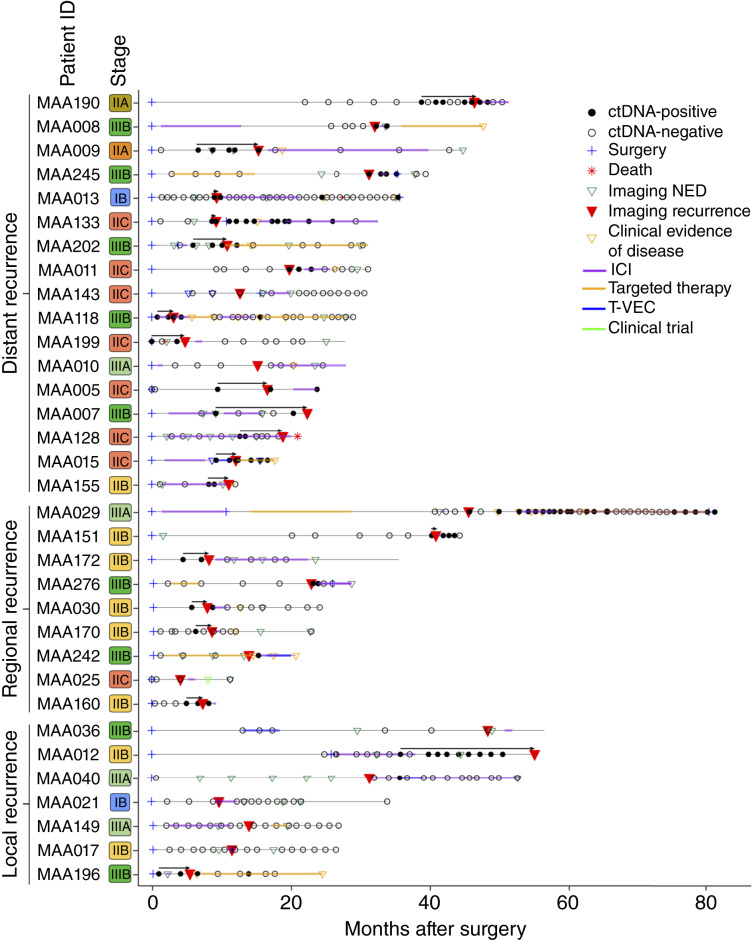
Overview plot showing longitudinal ctDNA status, treatment regimen, and clinical outcomes for patients with stages I–IIIb melanoma who had radiographic recurrence after surgery. Arrows represent time from the first ctDNA-positive result to recurrence. NED, no evidence of disease; T-VEC, talimogene laherparepvec.

In total, 1,578 plasma samples were collected from 190 patients [median age: 64 years (range: 25–89 years)] with stage I to IIIB melanoma (stage IA: 2.1%, *n* = 4; stage IB: 5.8%, *n* = 11; stage IIA: 7.4%, *n* = 14; stage IIB: 33.7%, *n* = 64; stage IIC: 13.7%, *n* = 26; stage IIIA: 14.7%, *n* = 28; stage IIIB: 22.6%, *n* = 43). A median of 7 tests (range: 1–33) per patient were performed over a median period of 24.6 months (range: 3.7–74.7 months). Study cohort demographics with regard to tumor site, melanoma subtype, mutational status, source of tissue used to build the assay, site of recurrence, and treatment received are described in Table 1. The complete clinical course for all patients who experienced recurrence (*N* = 33) is depicted in Fig. 2. Additionally, the complete clinical course for all patients, stratified by stage, is shown in Supplementary Fig. S1–S6.

**Table 1.** Study cohort demographics.

Characteristic (*n*, %)	*N* = 190	Characteristic (*n*, %)	*N* = 190
Age (median, range)	64 (25–89)	*BRAF* ^V600^ status	
Gender		Mutant	59 (31.1%)
Male	123 (64.7%)	Wild type	75 (39.5%)
Female	67 (35.3%)	Unknown	56 (29.5%)
Ethnicity		Source of tissue for test	
Black or African American	2 (1.1%)	Excisional biopsy	42 (22.1%)
Hispanic or Latino	5 (2.6%)	Punch biopsy	6 (3.2%)
White	183 (96.3%)	Shave biopsy	86 (45.3%)
AJCC eighth edition stage		Surgical resection	56 (29.5%)
IA	4 (2.1%)	Recurrence	
IB	11 (5.8%)	Yes	33 (17.4%)
IIA	14 (7.4%)	No	157 (82.6%)
IIB	64 (33.7%)	Sites of recurrence (% of all recurrences)	
IIC	26 (13.7%)	Local	7 (21.2%)
IIIA	28 (14.7%)	Regional	9 (27.3%)
IIIB	43 (22.6%)	Distant	17 (51.5%)
Site of primary location		Adjuvant treatment	
Head and neck	52 (27.4%)	Yes	83 (43.7%)
Lower extremity	32 (16.8%)	No	107 (56.3%)
Trunk	64 (33.7%)	Adjuvant therapy type	
Upper extremity	37 (19.5%)	Immunotherapy	66 (79.5%)
Unknown	5 (2.6%)	Targeted therapy	12 (14.5%)
Subtype		Radiotherapy	4 (4.8%)
Nodular	61 (32.1%)	Talimogene laherparepvec	1 (1.2%)
Superficial spreading	57 (30.0%)	​	​
Other	33 (17.4%)	​	​
Unknown	39 (20.5%)	​	​

### Longitudinal ctDNA status is associated with RFS outcomes in patients with stage I to IIIB melanoma

Among 33 patients who relapsed, 72.7% (*n* = 24/33) were ctDNA-positive prior to or at the time (±2 weeks) of recurrence, with a specificity of 94.9% (*n* = 149/157). In patients with distant/regional relapse, 84.6% (*n* = 22/26) were ctDNA-positive prior to recurrence, with a specificity of 93.9% (*n* = 154/164) in this group. Excluding patients who were treated on molecular recurrence (*N* = 3) and patients with less than 6 months of follow-up after a ctDNA-positive result (*N* = 2), serial ctDNA negativity was observed in 97.4% (*n* = 149/153) of patients who did not relapse. The median lead time between the first ctDNA-positive result and clinical or radiographic recurrence was 3.3 months (range: 0.3–17.1 months; Supplementary Fig. S7). To address potential fluctuations in ctDNA results, we also recalculated lead time using the last ctDNA-positive sample prior to imaging-confirmed recurrence, excluding intervening negative results. Using this definition, the median lead time was 2.1 months (range: 0.3–13.5 months). These complementary analyses demonstrate that ctDNA detection consistently preceded radiographic recurrence by several months, regardless of the analytic approach. Among 33 patients with recurrence and an available first postoperative ctDNA result, 27 were ctDNA-negative at the initial draw. Of these, 18 (66.7%) subsequently converted to ctDNA-positive prior to recurrence, whereas 9 (33.3%) remained ctDNA-negative through recurrence. Of the remaining 9 patients who were ctDNA-negative prior to or at the time of recurrence, 2 turned ctDNA-positive after recurrence (1 local and 1 regional) and 7 remained negative by the time of data cutoff (4 local recurrences, 2 distant, and 1 regional).

**Figure 3.**
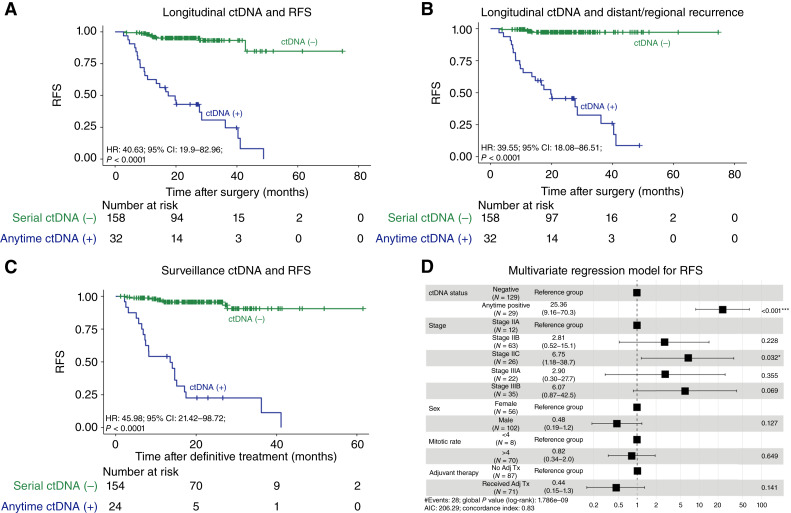
Association between ctDNA and RFS. **A,** Kaplan–Meier estimates for RFS stratified by longitudinal ctDNA status. Patients who experienced anytime ctDNA positivity demonstrated shorter RFS compared with patients with serially negative ctDNA. **B,** Kaplan–Meier estimates for distant/regional RFS stratified by longitudinal ctDNA status. ctDNA positivity at any postoperative timepoint was associated with significantly inferior distant/regional RFS compared with patients with serially negative ctDNA. **C,** Kaplan–Meier estimates for RFS stratified by ctDNA status during post–definitive treatment surveillance. ctDNA positivity during post–definitive treatment surveillance was associated with inferior RFS when compared with patients who were serially ctDNA-negative. **D,** The unadjusted HRs (squares) and 95% CIs (horizontal lines) are shown for each prognostic factor. The vertical dotted line represents the null hypothesis. As shown, anytime ctDNA-positive was the most prognostic factor for RFS compared with all other factors. Adj Tx, adjuvant therapy, AIC, Akaike Information Criterion.

Patients who experienced ctDNA positivity at any postoperative timepoint experienced shorter RFS (HR: 40.63; 95% CI, 19.9–82.96; *P* < 0.0001; Fig. 3A) compared with patients who were serially ctDNA-negative. Specifically, distant/regional RFS was significantly shorter in individuals with anytime ctDNA positivity [HR: 39.55; 95% CI, 18.08–86.5; *P* < 0.0001; Fig. 3B). When restricting to the post–definitive treatment surveillance analysis, including in patients who either completed or never received standard adjuvant therapy, we found that patients who showed ctDNA positivity during post–definitive treatment surveillance experienced significantly inferior RFS (HR: 45.98; 95% CI, 21.42–98.72; *P* < 0.0001; Fig. 3C). Multivariate analysis confirmed ctDNA positivity any time after surgery to be the most significant prognostic factor associated with RFS when compared with other clinicopathologic factors such as stage, sex, and mitotic rate (HR: 25.36; 95% CI, 9.16–70.3; *P* < 0.001; Fig. 3D). In stage-stratified analyses, anytime ctDNA positivity remained strongly associated with inferior RFS in both stage II (HR: 60.15; 95% CI, 20.13–179.8; *P* < 0.0001; Supplementary Fig. S8A) and stage III melanoma (HR: 30.71; 95% CI, 6.08–155; *P* < 0.0001; Supplementary Fig. S8B), further supporting the robustness of ctDNA as a prognostic biomarker across homogeneous subgroups.

**Figure 4.**
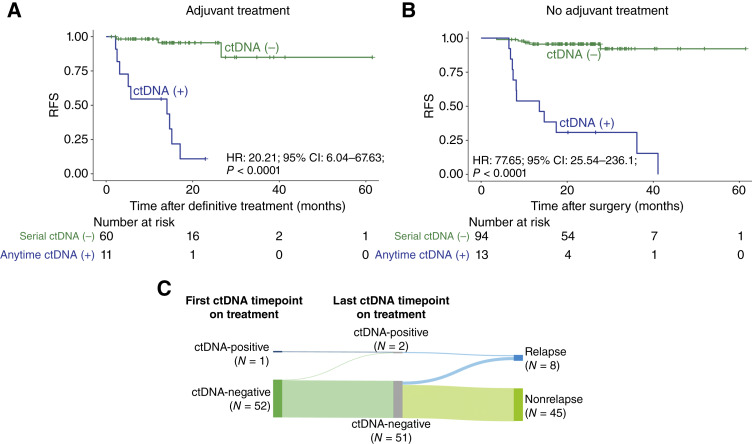
**A,** Kaplan–Meier RFS curves stratified by ctDNA status among patients who received adjuvant systemic therapy. Anytime ctDNA positivity was associated with significantly inferior RFS. **B,** Kaplan–Meier RFS curves stratified by ctDNA status among patients who did not receive adjuvant therapy, showing a similar strong association between ctDNA positivity and recurrence. **C,** Sankey diagram illustrating ctDNA dynamics after systemic therapy initiation among 53 patients with ≥1 on-treatment ctDNA timepoint. Of the 8 patients who recurred, 2 exhibited persistent ctDNA positivity or converted from ctDNA-negative to ctDNA-positive despite treatment. A subset demonstrated transient clearance before recurrence.

Moreover, for patients who received adjuvant therapy, ctDNA positivity at any timepoint after adjuvant therapy was associated with inferior RFS (HR: 20.21; 95% CI, 6.04–67.63; *P* < 0.0001; Fig. 4A). This was also observed in patients with ctDNA positivity who underwent surgery and did not receive adjuvant treatment (HR: 77.65; 95% CI, 25.54–236.1; *P* < 0.0001; Fig. 4B). We also assessed ctDNA dynamics after systemic therapy initiation. Among 53 patients with ≥1 on-treatment ctDNA timepoint, only 8 experienced recurrence during follow-up, allowing an exploratory assessment of molecular response patterns. As shown in the Sankey diagram (Fig. 4C), relapse was observed in patients who remained ctDNA-positive or converted from ctDNA-negative to ctDNA-positive despite treatment (2 patients) and in an additional subset (6 patients) who demonstrated transient molecular clearance before recurrence.

### Impact of ctDNA on clinical management of patients with melanoma

**Figure 5.**
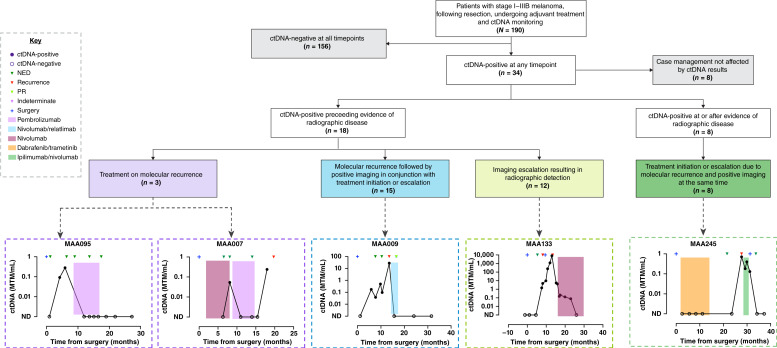
Flow diagram depiction of ctDNA-driven changes in clinical management with representative case examples. Of the 190 patients included in our study, 34 had ctDNA positivity at any time point. Of these, clinical care was influenced by ctDNA positivity in 26 cases, ranging from imaging escalation to treatment initiation, switch, or escalation. MTM, mean tumor molecules; ND, not detected; NED, no evidence of disease; PR, partial response.

We evaluated how ctDNA testing influenced clinical decision-making in this real-world cohort. Among the 190 patients included in the analysis, 34 had at least one positive ctDNA test result during the course of their clinical care. In 76.5% (*n* = 26/34) of these patients, ctDNA positivity was associated with a change in clinical management. These changes ranged from imaging escalation to treatment initiation, switch, or escalation. Figure 5 illustrates these various clinical scenarios.

In the first clinical scenario, we identified cases where a “treat-on-molecular recurrence” approach was employed, in which systemic therapy was initiated based on ctDNA positivity without any radiographic evidence of disease. Of 18 patients with a ctDNA-positive test result preceding imaging findings, 3 received systemic therapy solely because of the presence of ctDNA as an indicator of MRD. These 3 patients cleared ctDNA after initiation of systemic therapy. Of these, 2 patients persistently cleared and remained disease-free at the time of data cutoff. This includes 1 patient with stage IIA disease (MAA095) who was treated with pembrolizumab after a serial increase in ctDNA levels despite negative imaging; this patient cleared their ctDNA after treatment with pembrolizumab, and remained disease-free. In contrast, 1 patient (MAA007) had transient clearance on adjuvant immunotherapy followed by a ctDNA-detected brain recurrence with a 1.8-month lead time over imaging (Fig. 5).

In a second clinical scenario, imaging was intensified in response to a positive ctDNA result, which resulted in an earlier radiographic detection of disease in 12 patients. Furthermore, for 15 patients, the treatment was either started, changed, or increased after both ctDNA positivity and imaging-confirmed disease; 9 of these achieved radiographic and molecular clearance and remained disease-free at the time of data cutoff. For example, a patient (MAA009) with stage IIA disease developed increasing ctDNA levels during post–definitive treatment surveillance, which led to the discovery of radiographically confirmed liver metastasis. The patient was initiated on nivolumab/relatlimab and achieved a complete response on imaging. Similarly, patient MAA133 had stage IIC disease and positive ctDNA 8 months after surgery, preempting a biopsy-confirmed neck recurrence. Despite resection, ctDNA persisted, and later widespread metastases were found (hypermetabolic hepatic lesion, multifocal osseous metastases, multiple new hypermetabolic subcutaneous lesions, and a new hypermetabolic left lower lung nodule). The patient was initiated on single-agent nivolumab and responded well per clinical assessment.

The third clinical scenario involved 8 patients with ctDNA positivity concurrent with or following radiographic disease. All 8 patients had treatment initiated, switched, or escalated after both ctDNA and imaging-confirmed disease. For example, patient MAA245, with stage IIIB disease, had simultaneous ctDNA positivity and recurrence over 2 years after surgery. This patient received one cycle of immunotherapy before resection and remained disease-free at the time of data cutoff, with ctDNA clearance aligning with their clinical course.

## Discussion

In this real-world cohort of patients with stage I to IIIB melanoma, we demonstrate that ctDNA positivity is a strong and independent prognostic marker of poor clinical outcomes. Longitudinal ctDNA monitoring was significantly associated with RFS, and ctDNA positivity emerged as the strongest prognostic factor in multivariate analysis. Additionally, ctDNA negativity during post–definitive treatment surveillance was strongly associated with improved RFS, underscoring the clinical impact of serial ctDNA assessment in the postsurgical setting. These findings contribute real-world evidence supporting the role of ctDNA as a valuable biomarker for risk stratification in nonmetastatic melanoma.

A defining strength of this study is the detailed characterization of clinical scenarios in which ctDNA results directly altered management. Among ctDNA-positive patients, nearly three-quarters experienced a change in care, ranging from intensified imaging to treatment initiation, switch, or escalation. In a subset, systemic therapy was started on the basis of ctDNA detection alone, before radiographic evidence of disease, representing a treat-on-molecular recurrence approach. To the best of our knowledge, this is the first time such real-world, ctDNA-guided intervention examples have been reported in melanoma. In these cases, molecular clearance following therapy was observed, with durable disease-free status in some patients, underscoring the potential of ctDNA to provide a therapeutic window for early intervention. Although a subset of patients demonstrated ctDNA clearance after initiation of adjuvant or systemic therapy, the small number of evaluable cases precluded robust statistical comparisons between ctDNA-positive and ctDNA-negative groups by adjuvant treatment status. This remains an important area for future investigation, ideally within prospective trials designed to test ctDNA-guided adjuvant therapy selection.

These observations align with studies demonstrating the prognostic value of ctDNA in melanoma across various disease stages (1, 17–21). Several studies have reported the value of ctDNA detection in the setting of advanced melanoma (16, 19–21). A previous study demonstrated the feasibility of ctDNA detection and treatment monitoring using a personalized, tumor-informed ctDNA assay in the setting of stage III to IV melanoma (1). Here, we confirm feasibility of the same methodology within the setting of surgically resectable (stage I–IIIB) melanoma. Notably, greater than 70% of tests were generated from WES of biopsy tissue. We found ctDNA, when analyzed longitudinally, to be prognostic of RFS. Moreover, in multivariate analysis, we found ctDNA positivity to be the strongest prognostic factor in our cohort, which was independently associated with poorer RFS. In the post–definitive treatment surveillance setting, ctDNA negativity was highly associated with improved RFS (HR: 45.98; *P* < 0.001), indicative of the prognostic value of serial monitoring in the observation or off-treatment setting.

Our findings build on existing literature supporting the prognostic value of ctDNA in melanoma. In a retrospective analysis of 161 patients with stage III melanoma, Lee and colleagues found the detection of *BRAF/NRAS*-mutant ctDNA by digital droplet PCR at a single timepoint 12 weeks after surgery to be associated with poor disease-free interval (HR: 3.12; 95% CI, 1.79–5.47; *P* < 0.0001) and inferior distant metastasis-free interval (HR: 3.22; 95% CI, 1.80–5.79; *P* < 0.0001; ref. 17). Similarly, using targeted amplicon-based next-generation sequencing or pyrosequencing, Tan and colleagues (18) found that detectable ctDNA within 12 weeks after surgery was a significant risk factor for relapse (RFS HR: 10.0; 95% CI, 4.3–24; *P* < 0.001). Moreover, they found that ctDNA status remained an independent predictor of outcomes in multivariate analyses after adjusting for disease stage and *BRAF* mutation status (18). McEvoy and colleagues report similar findings in a proof-of-concept study based on three cases (2 patients with stage I and 1 patient with stage III) with confirmed disease recurrence (22).

A notable finding from our analysis involves the use of ctDNA to guide treatment initiation in the absence of radiographic evidence of disease—an approach referred to as “treat-on-molecular recurrence.” In a subset of 18 patients for whom ctDNA detection preceded imaging findings, 3 patients were initiated on systemic therapy based solely on ctDNA positivity. All 3 demonstrated subsequent ctDNA clearance following treatment, with 2 remaining disease-free at the time of data cutoff. These observations suggest that intervening at the molecular level prior to radiographic recurrence may offer a window of opportunity for early therapeutic intervention and potentially improved disease control. Although these findings are encouraging, additional data from prospective studies are needed to draw definitive conclusions about whether early intervention based on ctDNA confers improved outcomes.

Several clinical scenarios merit prospective evaluation of ctDNA-guided management. For example, patients with comorbidities or autoimmune conditions who face high risks of immune-related complications might safely defer adjuvant therapy unless ctDNA becomes detectable, adopting a treat-on-molecular recurrence approach. Notably, ctDNA-triggered initiation of adjuvant immunotherapy has shown clinical benefit in other solid tumors, such as muscle-invasive bladder cancer in the phase III IMvigor011 trial, in which ctDNA-guided atezolizumab significantly improved disease-free and OS compared with placebo (23). Conversely, serial ctDNA monitoring may help identify individuals with low-stage (I–IIA) melanoma who could benefit from early systemic intervention. Collectively, these scenarios underscore the potential of ctDNA to personalize surveillance and treatment strategies in resected melanoma, a hypothesis best addressed through future biomarker-guided interventional trials.

Our study has limitations inherent to its retrospective and real-world design, including patient selection bias, variability in the timing and frequency of plasma collection, and heterogeneity in adjuvant therapy and surveillance imaging. To mitigate potential guarantee-time bias arising from variable sampling intervals, ctDNA status was modeled as a time-varying covariate in Cox analyses, allowing each patient’s risk status to update with every ctDNA result and preserving the temporal relationship between ctDNA detection and recurrence. Patients were managed across multiple institutions with nonstandardized follow-up practices, which, while reflecting real-world clinical diversity, may introduce variability that could confound our findings. Given the heterogeneity in timing of postoperative blood draws, our findings most robustly characterize ctDNA as a post–definitive treatment surveillance biomarker, capable of providing real-time clinical decision support, as demonstrated by the diverse ctDNA-driven management scenarios shown in Fig. 5. Future prospective studies incorporating standardized early postoperative sampling are warranted to define ctDNA’s sensitivity for true MRD detection and its potential role in guiding adjuvant therapy decisions. Finally, our cohort spanned a wide range of disease stages (I–IIIB), and although individual stage subgroups were small, ctDNA positivity remained strongly associated with recurrence across stages, supporting its broad prognostic relevance.

In summary, this work demonstrates that longitudinal ctDNA monitoring in resected stage I to IIIB melanoma is prognostic and can meaningfully influence clinical management, including in cases without radiographic evidence of disease. These results echo the ability to add ctDNA monitoring to earlier stage (IA, IB, and IIA) melanoma for which no systemic interval surveillance imaging is recommended, even when only the initial biopsy sample is available. These findings, together with growing evidence from other solid tumors, support the potential integration of tumor-informed ctDNA testing into postsurgical surveillance paradigms. Prospective, biomarker-guided intervention trials remain essential to determine whether early treatment based on ctDNA detection can translate into improved long-term outcomes for patients with high-risk, nonmetastatic melanoma.

## Supplementary Material

Supplementary Figure 1 Overview plot of patients with stage IA/B melanoma.

Supplementary Figure 2 Overview plot of patients with stage IIA melanoma.

Supplementary Figure 3 Overview plot of patients with stage IIB melanoma.

Supplementary Figure 4 Overview plot of patients with stage IIC melanoma.

Supplementary Figure 5 Overview plot of patients with stage IIIA melanoma.

Supplementary Figure 6 Overview plot of patients with stage IIIB melanoma.

Supplementary Figure 7 Distribution of lead time between ctDNA detection and imaging-confirmed recurrence using the first ctDNA-positive sample prior to recurrence. Each dot represents an individual patient with green dots representing patients who became ctDNA-positive and whose initial post-positivity imaging remained negative with recurrence confirmed on subsequent imaging.

Supplementary Figure 8 Recurrence-free survival stratified by ctDNA status within Stage II and Stage III melanoma. (A) Kaplan–Meier recurrence-free survival (RFS) curves for patients with Stage II melanoma, stratified by longitudinal ctDNA status. Patients who were ctDNA-positive at any postoperative timepoint experienced significantly inferior RFS compared with those who remained serially ctDNA-negative throughout follow-up (p < 0.0001). (B) Kaplan–Meier RFS curves for patients with Stage III melanoma, similarly demonstrating markedly shorter RFS among patients who were ctDNA-positive at any postoperative timepoint relative to those with persistently negative ctDNA results (p < 0.0001).

Supplementary Table 1 Representativeness of the study participants

## Data Availability

The authors declare that all relevant, nonproprietary data used to conduct the analyses are available within the article. To protect the privacy and confidentiality of patients in this study, clinical data are not made publicly available in a repository or the Supplementary Material of the article but can be requested at any time from the corresponding author. All data shared will be de-identified.
