# Supplementary figures and images for: Longitudinal ctDNA Monitoring for Postsurgical Disease Surveillance in Patients with Stage I to IIIB Melanoma
Source: Clin Cancer Res. 2026 Feb 3;32(8):1513–21. doi: 10.1158/1078-0432.CCR-25-3643 (PMC13080317; doi:10.1158/1078-0432.CCR-25-3643)

## Slide 1
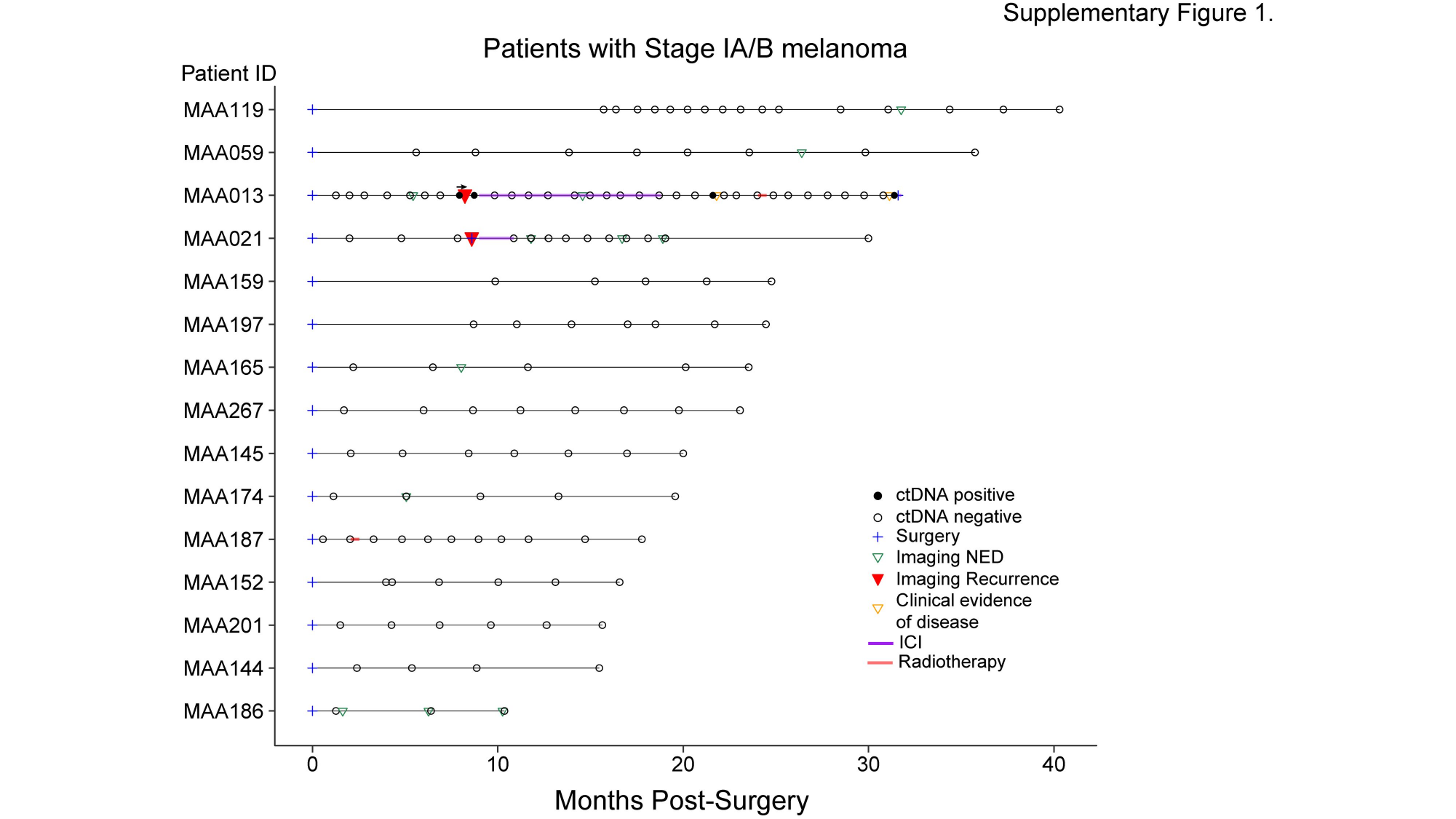

## Slide 2
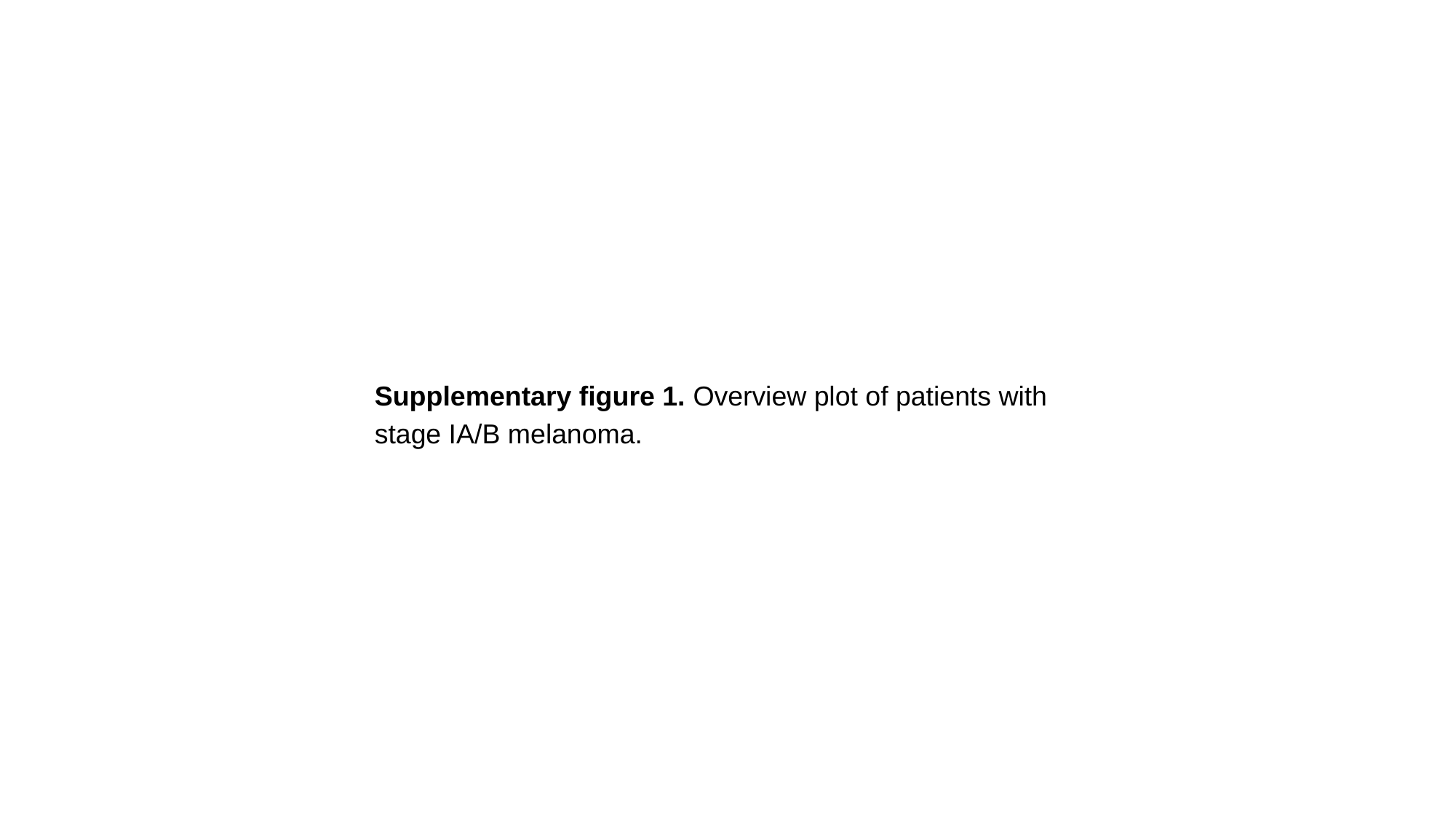

Supplementary figure 1. Overview plot of patients with stage IA/B melanoma.

Supplement: Supplementary Figure 1 — Overview plot of patients with stage IA/B melanoma. [file ccr-25-3643_supplementary_figure_1_suppfs1.pptx]

## Slide 1
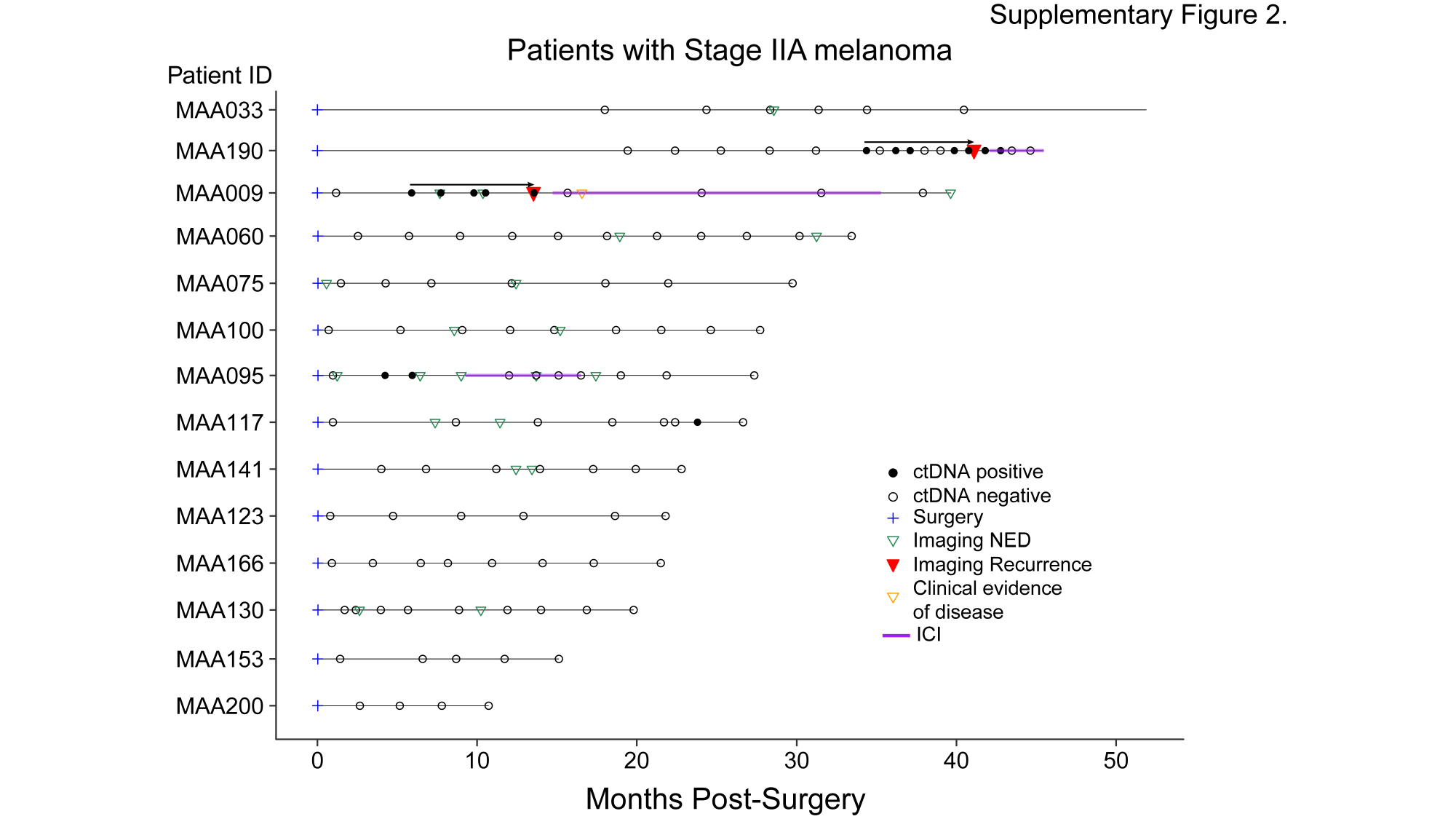

## Slide 2
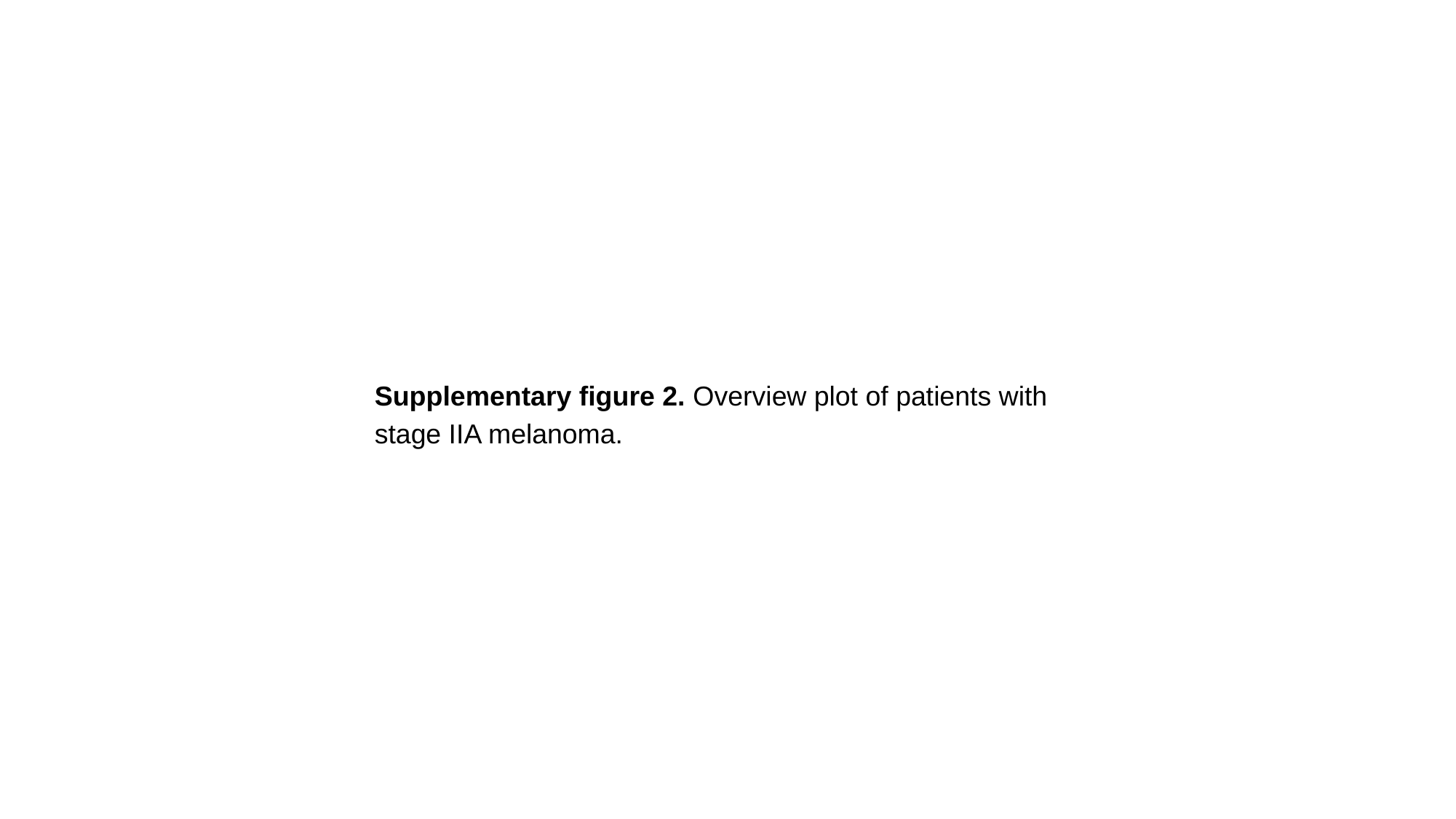

Supplementary figure 2. Overview plot of patients with stage IIA melanoma.

Supplement: Supplementary Figure 2 — Overview plot of patients with stage IIA melanoma. [file ccr-25-3643_supplementary_figure_2_suppfs2.pptx]

## Slide 1
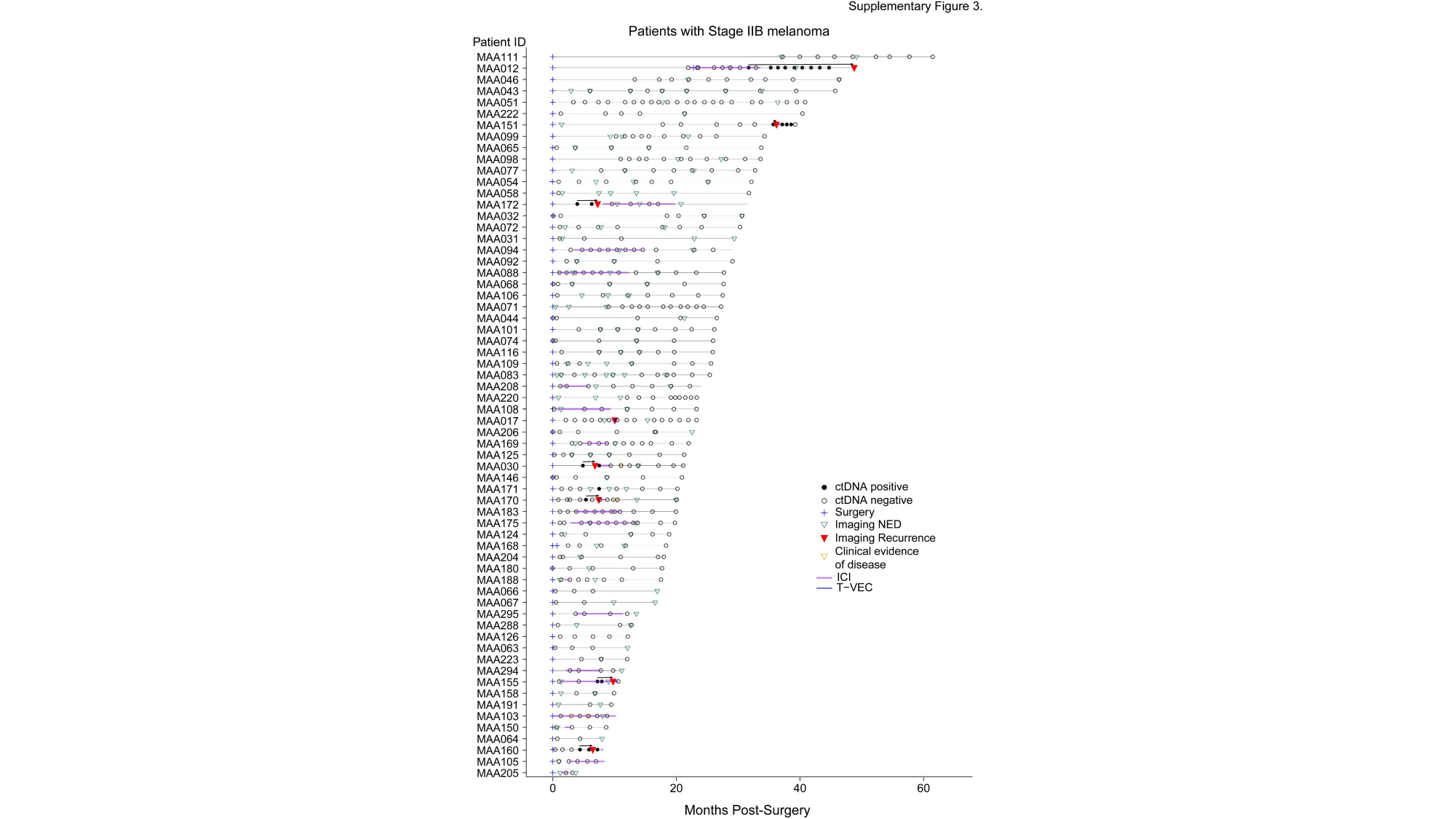

## Slide 2
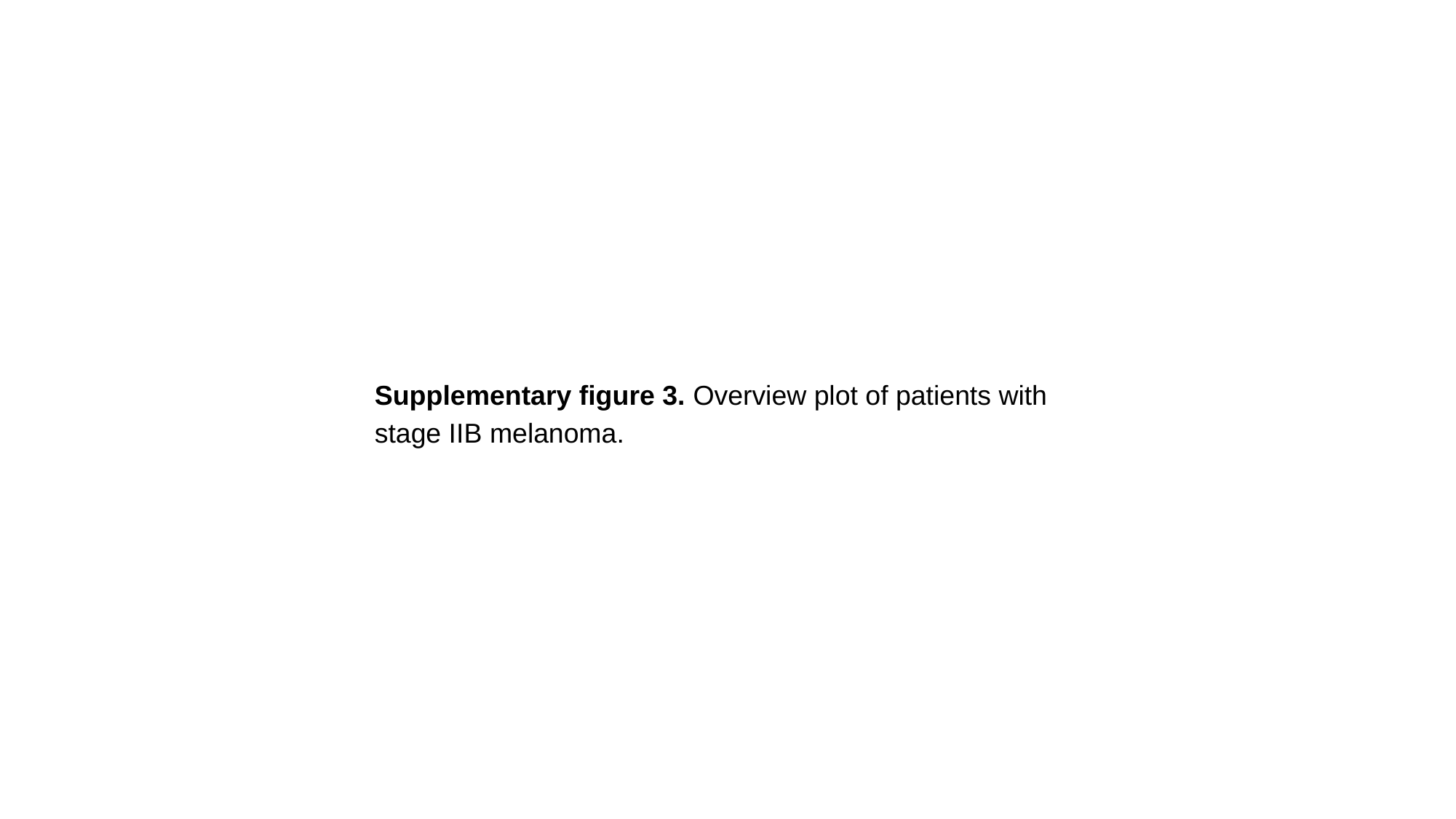

Supplementary figure 3. Overview plot of patients with stage IIB melanoma.

Supplement: Supplementary Figure 3 — Overview plot of patients with stage IIB melanoma. [file ccr-25-3643_supplementary_figure_3_suppfs3.pptx]

## Slide 1
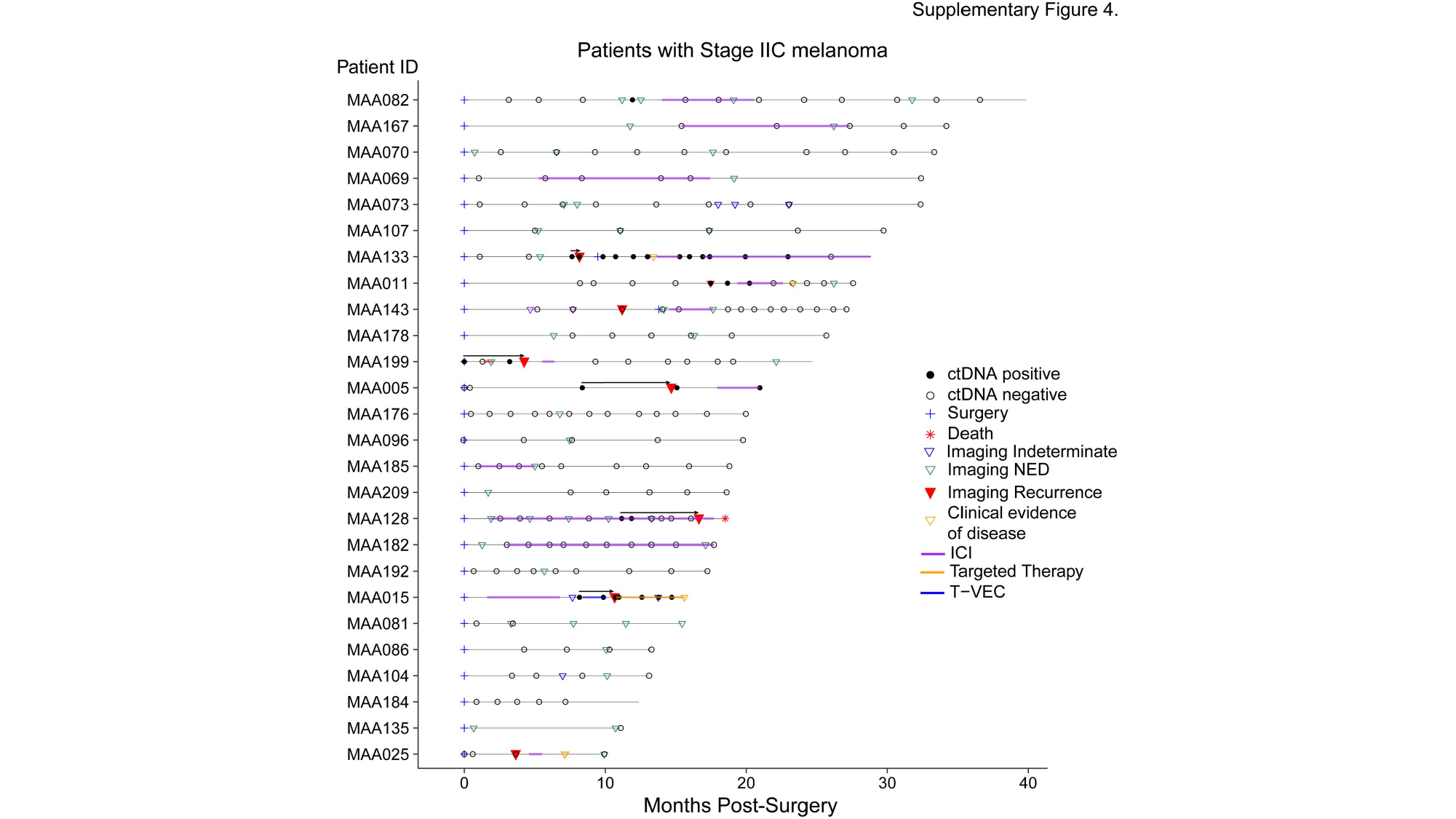

## Slide 2
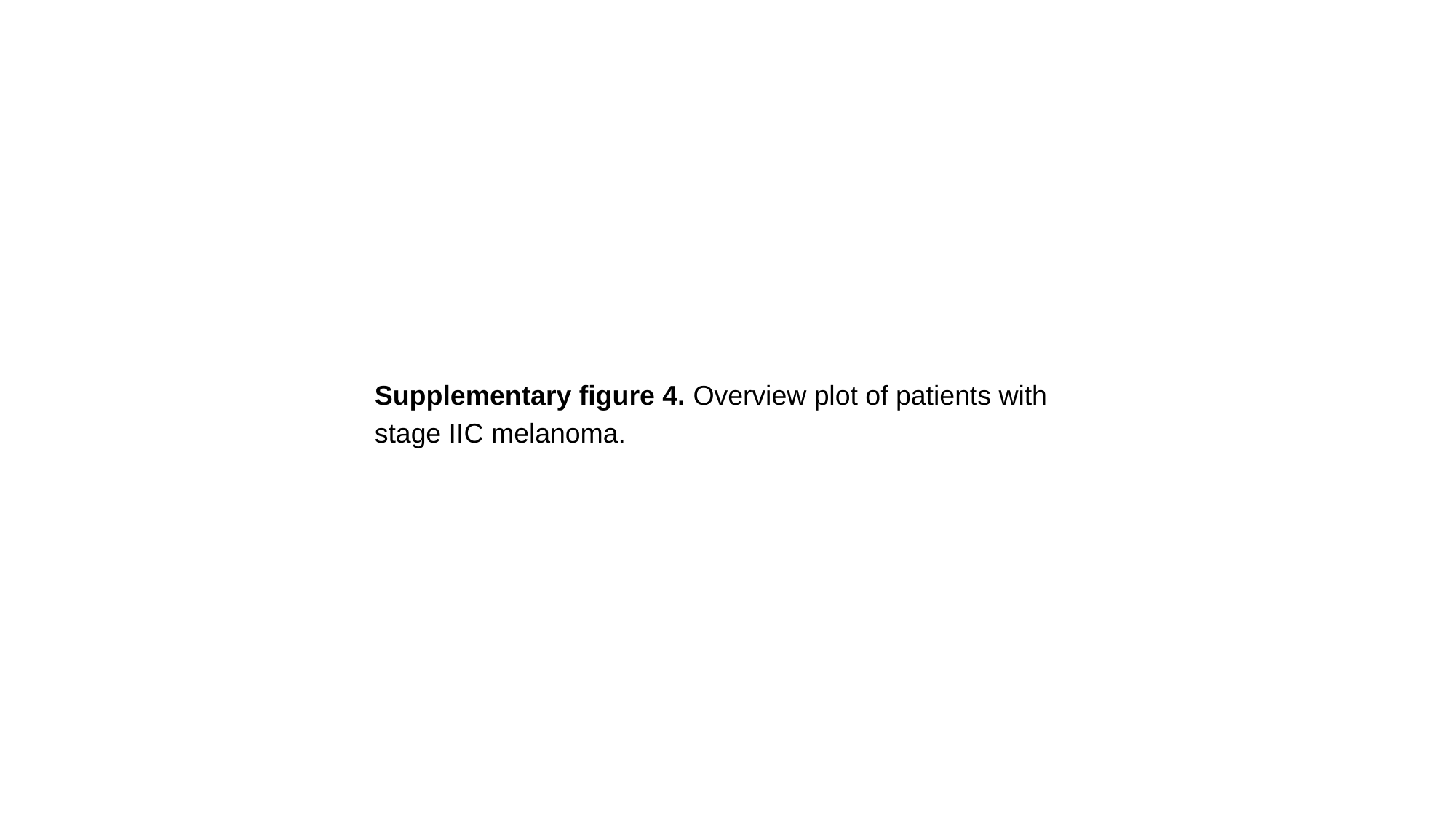

Supplementary figure 4. Overview plot of patients with stage IIC melanoma.

Supplement: Supplementary Figure 4 — Overview plot of patients with stage IIC melanoma. [file ccr-25-3643_supplementary_figure_4_suppfs4.pptx]

## Slide 1
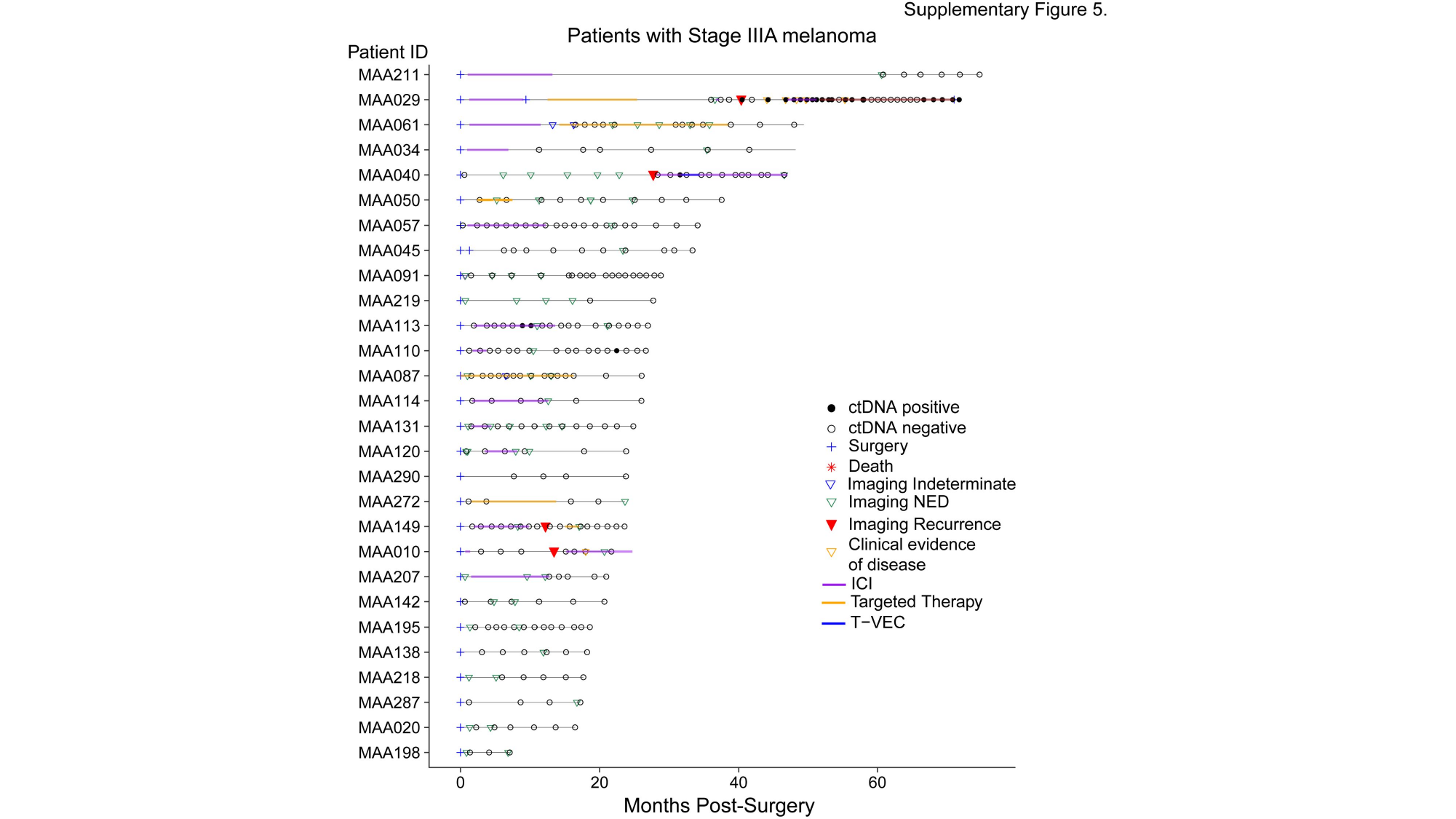

## Slide 2
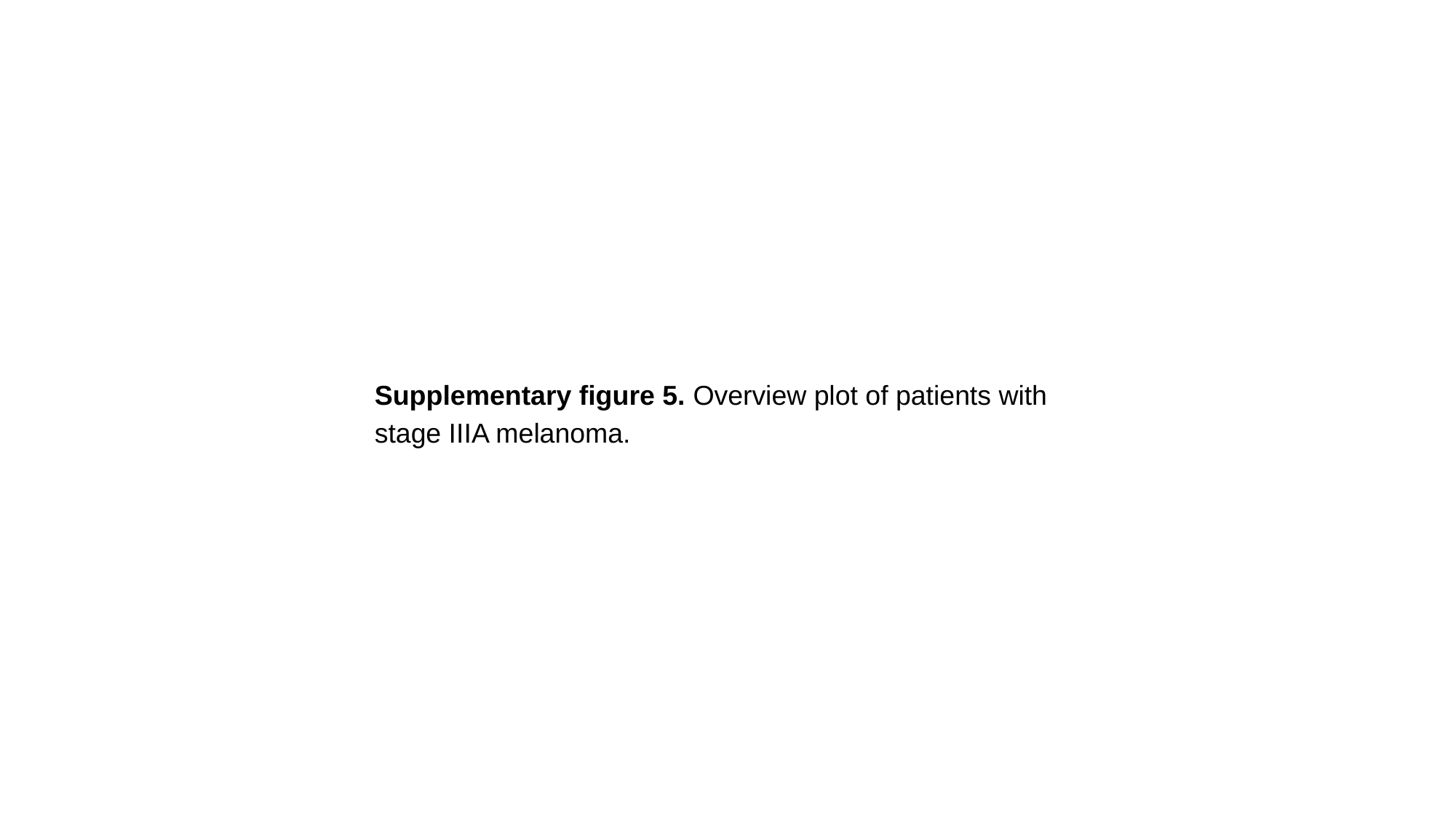

Supplementary figure 5. Overview plot of patients with stage IIIA melanoma.

Supplement: Supplementary Figure 5 — Overview plot of patients with stage IIIA melanoma. [file ccr-25-3643_supplementary_figure_5_suppfs5.pptx]

## Slide 1
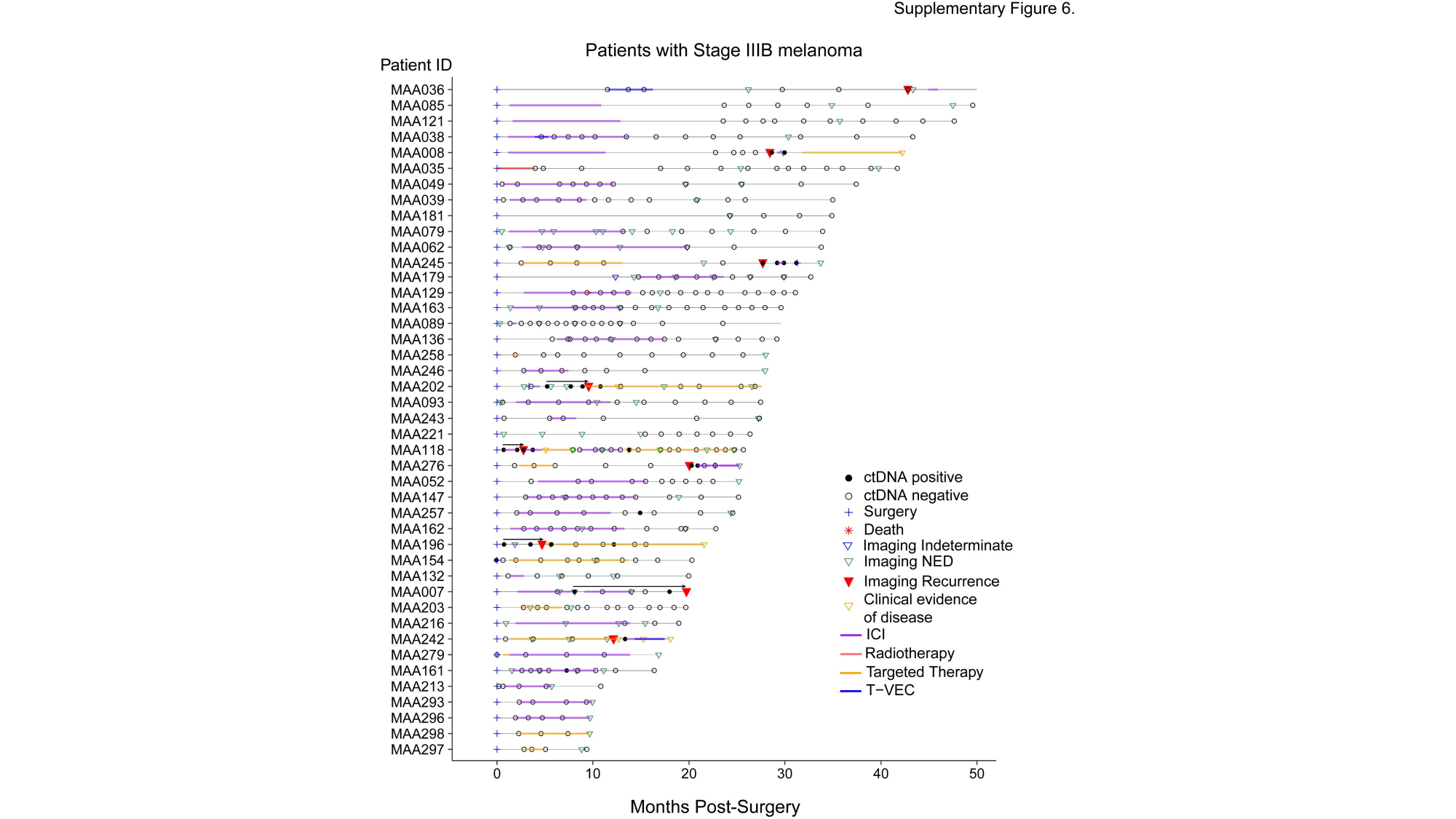

## Slide 2
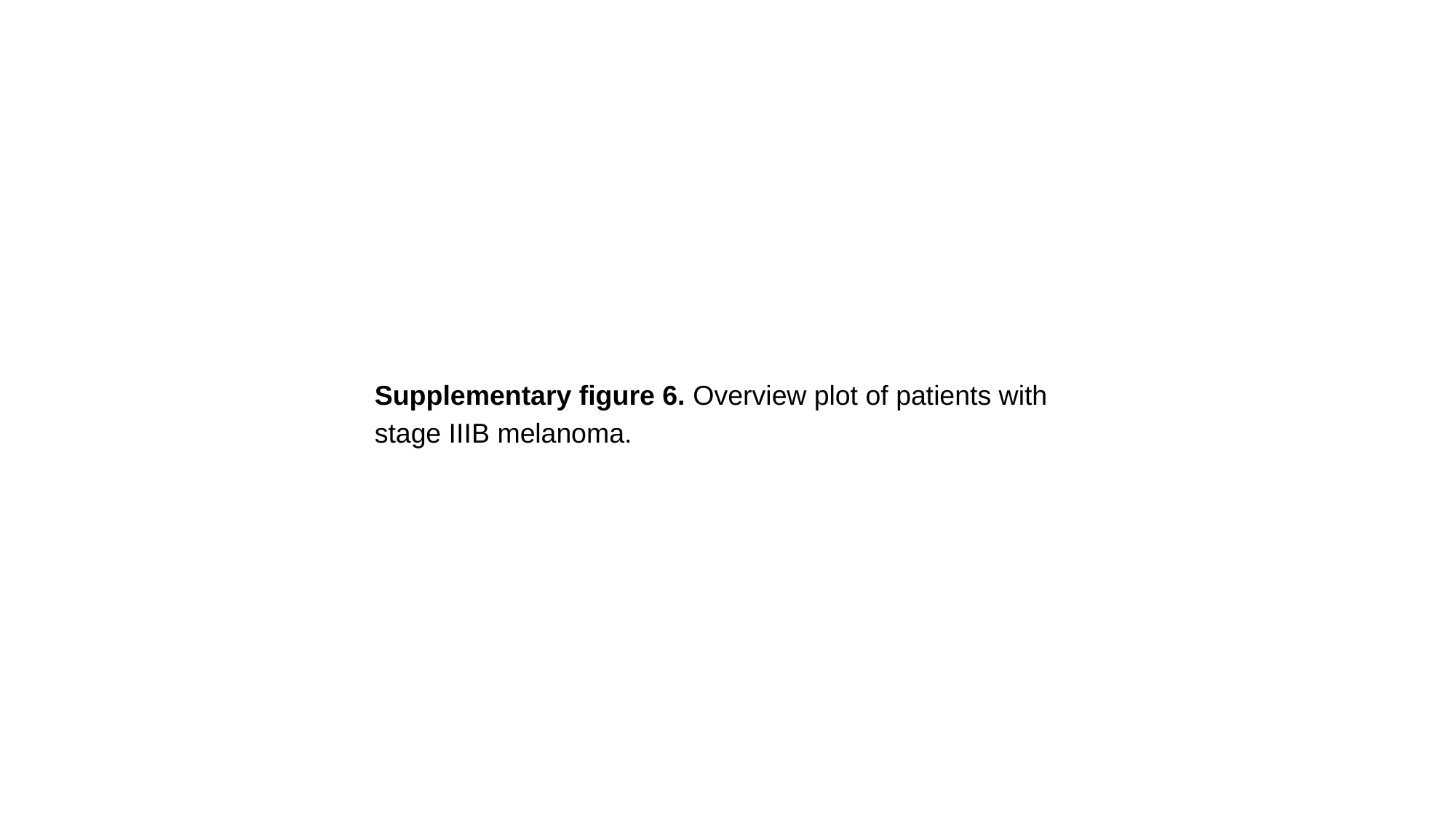

Supplementary figure 6. Overview plot of patients with stage IIIB melanoma.

Supplement: Supplementary Figure 6 — Overview plot of patients with stage IIIB melanoma. [file ccr-25-3643_supplementary_figure_6_suppfs6.pptx]

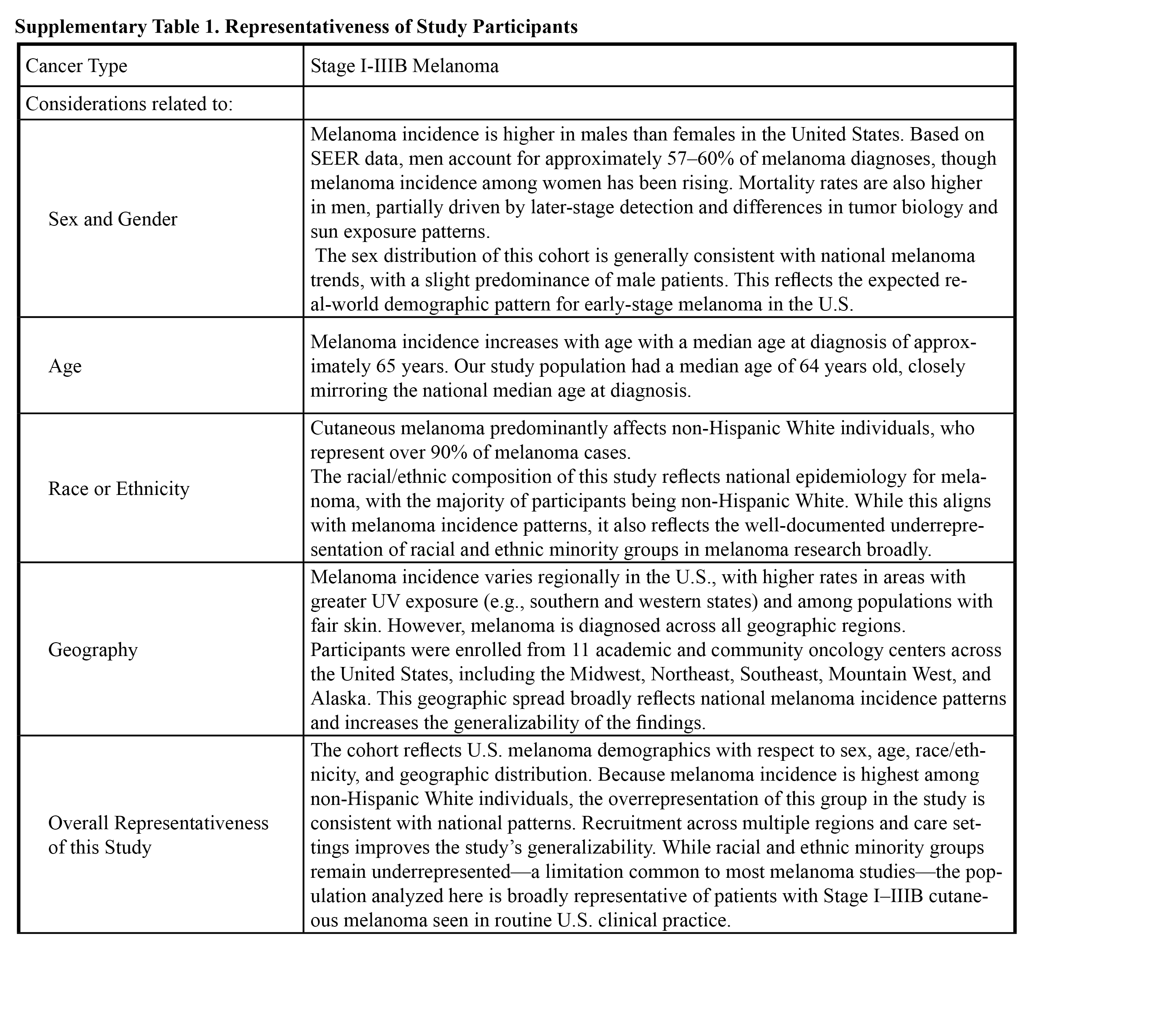

Supplement: Supplementary Table 1 — Representativeness of the study participants [file ccr-25-3643_supplementary_table_1_suppts1.png]
