# Supplementary material for: Longitudinal ctDNA Monitoring for Postsurgical Disease Surveillance in Patients with Stage I to IIIB Melanoma
Source: Clin Cancer Res. 2026 Feb 3;32(8):1513–21. doi: 10.1158/1078-0432.CCR-25-3643 (PMC13080317; doi:10.1158/1078-0432.CCR-25-3643)
Supplement: Supplementary Figure 7 — Distribution of lead time between ctDNA detection and imaging-confirmed recurrence using the first ctDNA-positive sample prior to recurrence. Each dot represents an individual patient with green dots representing patients who became ctDNA-positive and whose initial post-positivity imaging remained negative with recurrence confirmed on subsequent imaging. [file ccr-25-3643_supplementary_figure_7_suppfs7.pptx]

## Slide 1
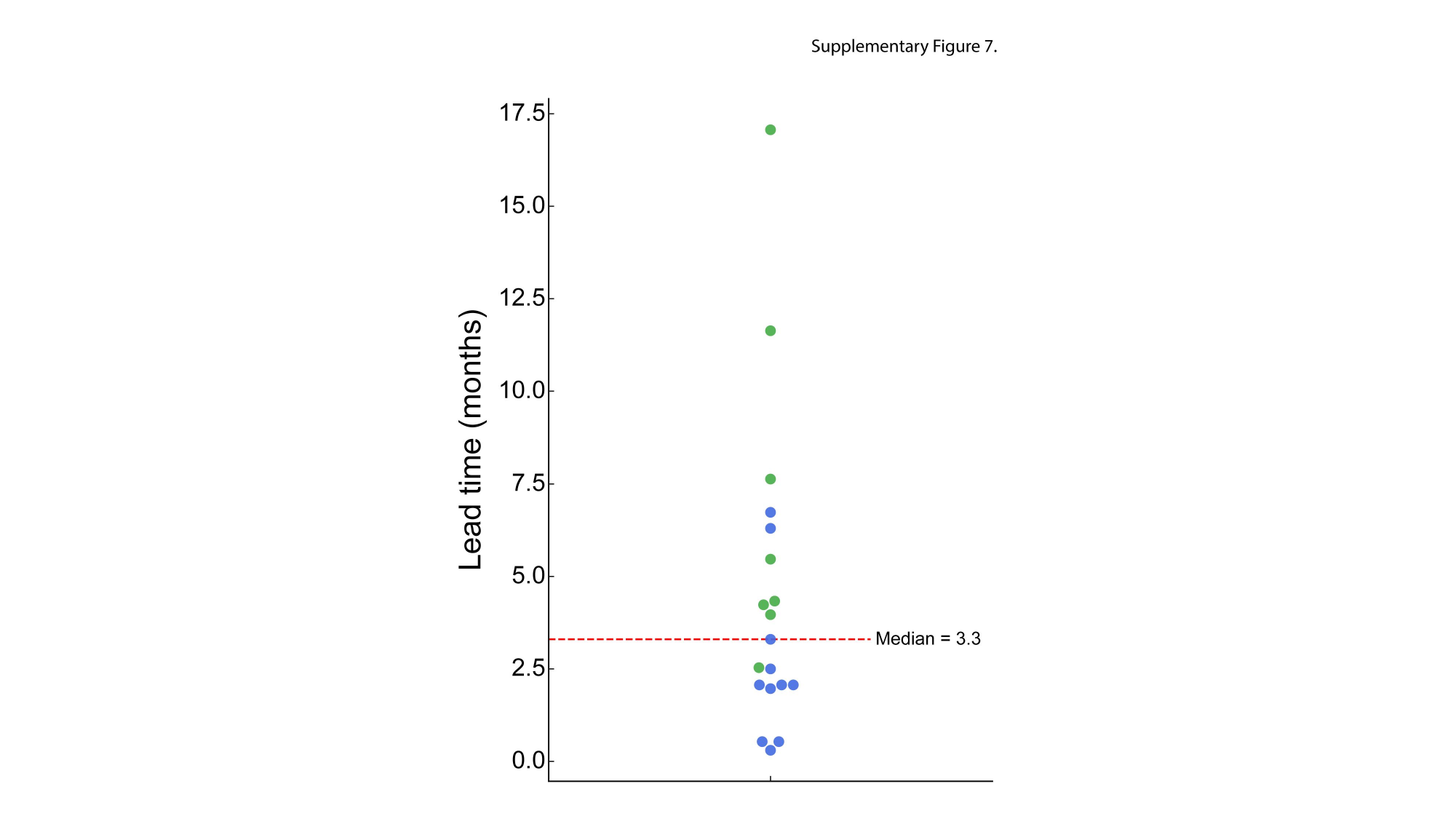

## Slide 2
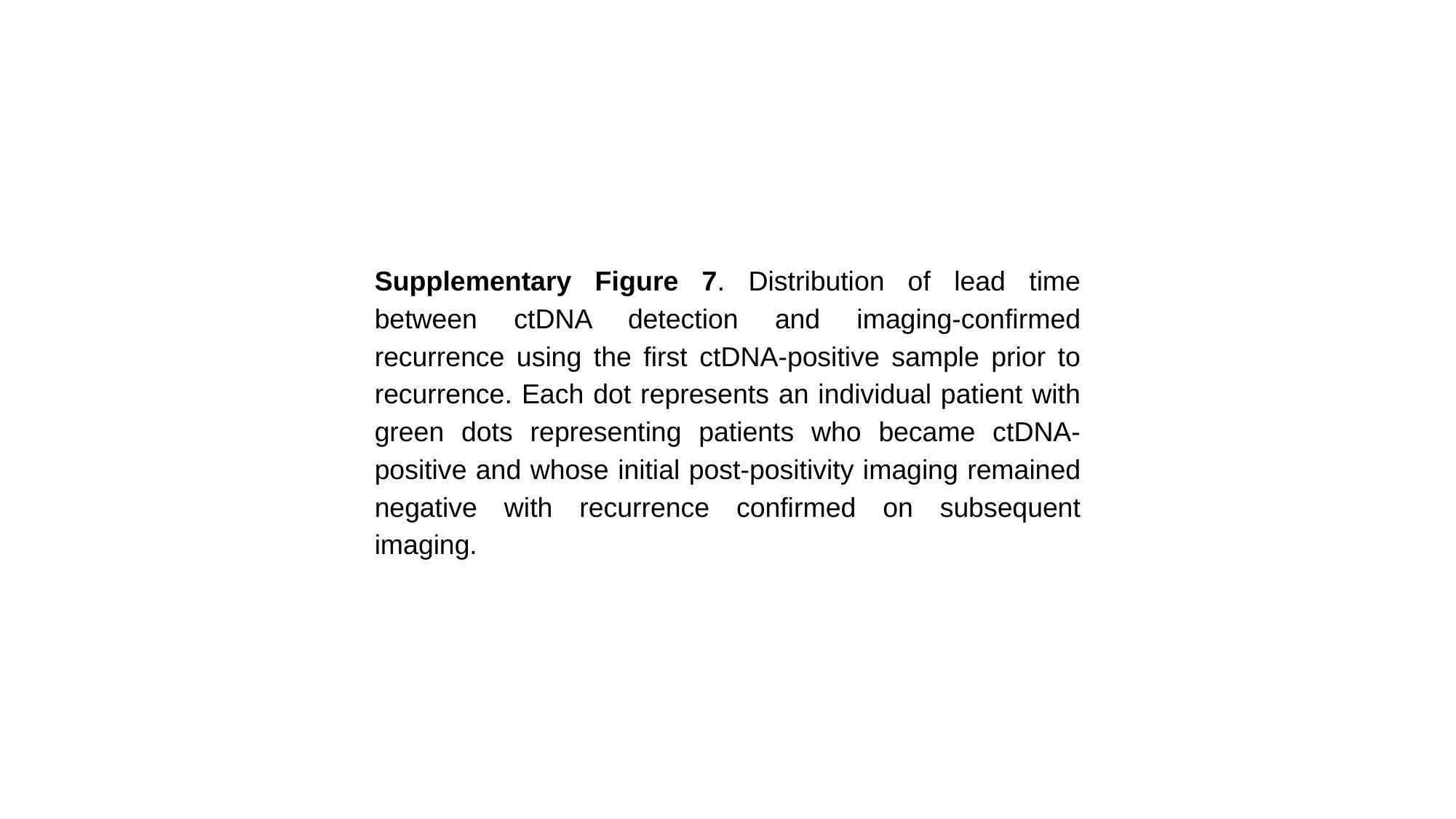

Supplementary Figure 7. Distribution of lead time between ctDNA detection and imaging-confirmed recurrence using the first ctDNA-positive sample prior to recurrence. Each dot represents an individual patient with green dots representing patients who became ctDNA-positive and whose initial post-positivity imaging remained negative with recurrence confirmed on subsequent imaging.
