# Supplementary material for: Longitudinal ctDNA Monitoring for Postsurgical Disease Surveillance in Patients with Stage I to IIIB Melanoma
Source: Clin Cancer Res. 2026 Feb 3;32(8):1513–21. doi: 10.1158/1078-0432.CCR-25-3643 (PMC13080317; doi:10.1158/1078-0432.CCR-25-3643)
Supplement: Supplementary Figure 8 — Recurrence-free survival stratified by ctDNA status within Stage II and Stage III melanoma. (A) Kaplan–Meier recurrence-free survival (RFS) curves for patients with Stage II melanoma, stratified by longitudinal ctDNA status. Patients who were ctDNA-positive at any postoperative timepoint experienced significantly inferior RFS compared with those who remained serially ctDNA-negative throughout follow-up (p < 0.0001). (B) Kaplan–Meier RFS curves for patients with Stage III melanoma, similarly demonstrating markedly shorter RFS among patients who were ctDNA-positive at any postoperative timepoint relative to those with persistently negative ctDNA results (p < 0.0001). [file ccr-25-3643_supplementary_figure_8_suppfs8.pptx]

## Slide 1
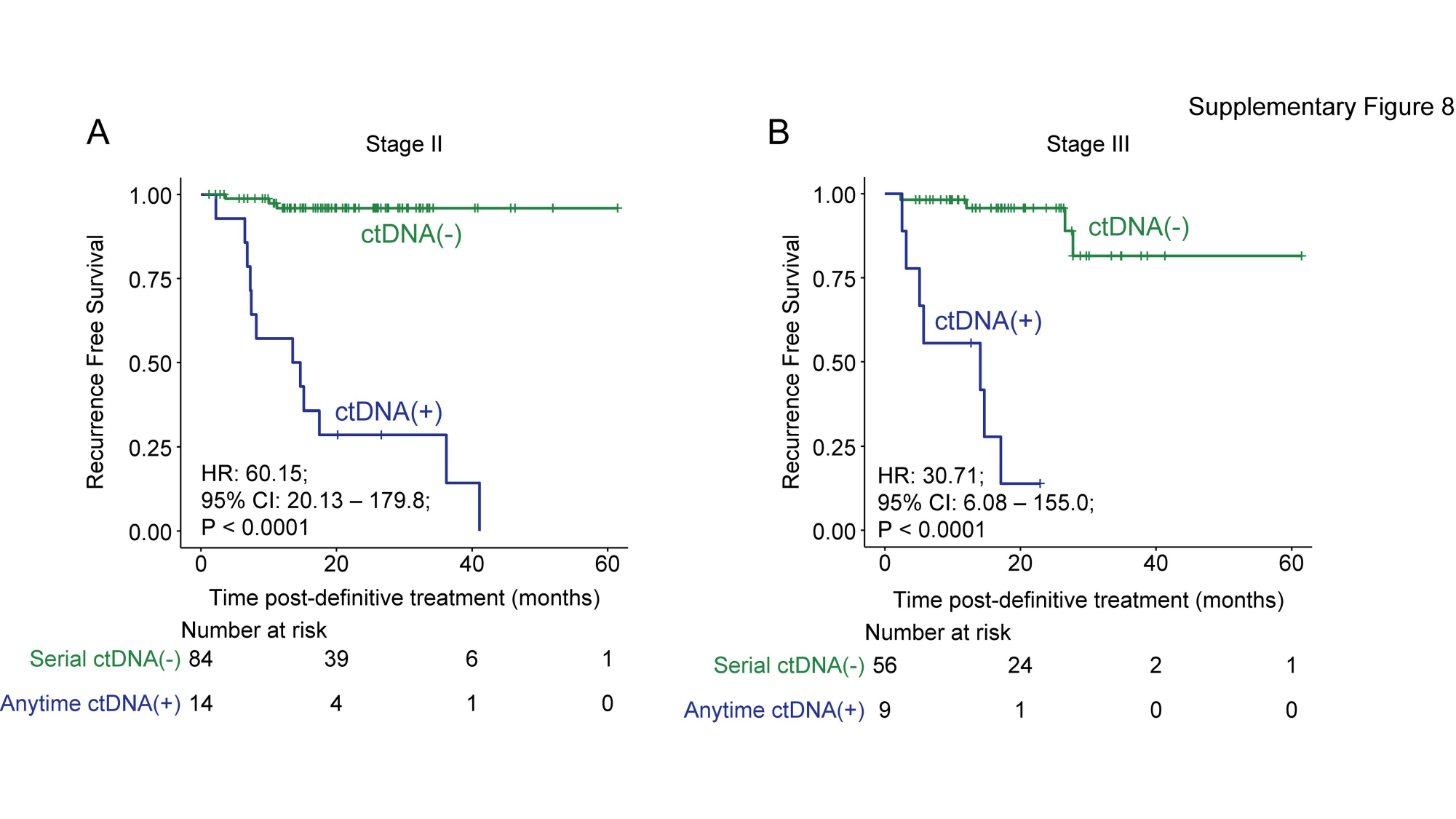

## Slide 2
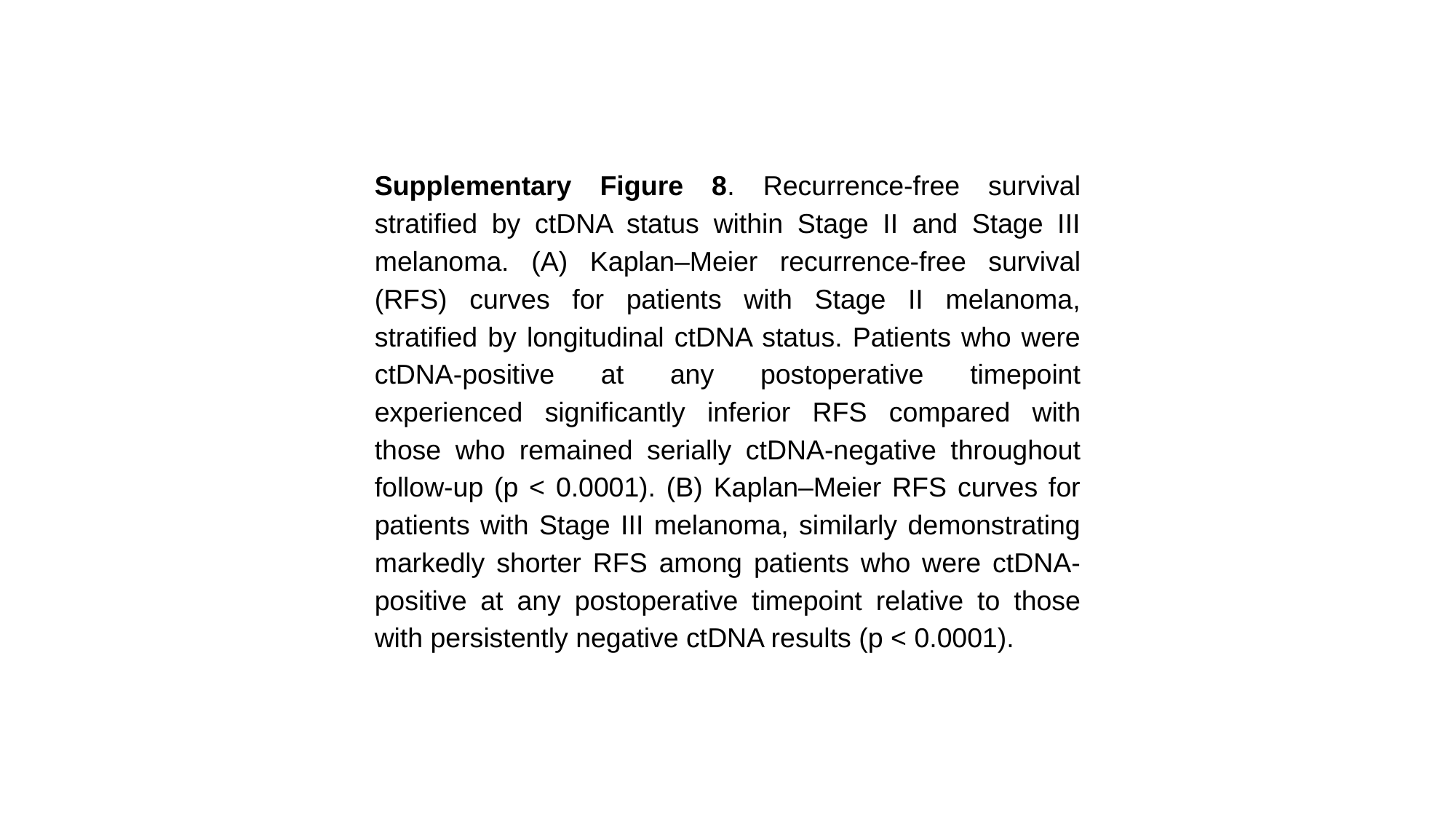

Supplementary Figure 8. Recurrence-free survival stratified by ctDNA status within Stage II and Stage III melanoma. (A) Kaplan–Meier recurrence-free survival (RFS) curves for patients with Stage II melanoma, stratified by longitudinal ctDNA status. Patients who were ctDNA-positive at any postoperative timepoint experienced significantly inferior RFS compared with those who remained serially ctDNA-negative throughout follow-up (p < 0.0001). (B) Kaplan–Meier RFS curves for patients with Stage III melanoma, similarly demonstrating markedly shorter RFS among patients who were ctDNA-positive at any postoperative timepoint relative to those with persistently negative ctDNA results (p < 0.0001).
